# Mixed venous oxygen tension is a crucial prognostic factor in pulmonary hypertension: a retrospective cohort study

**DOI:** 10.1186/s12890-022-02073-0

**Published:** 2022-07-20

**Authors:** Jun Nagata, Ayumi Sekine, Nobuhiro Tanabe, Yu Taniguchi, Keiichi Ishida, Yuki Shiko, Seiichiro Sakao, Koichiro Tatsumi, Takuji Suzuki

**Affiliations:** 1 Department of Respirology, Graduate School of Medicine, Chiba University, 1-8-1, Inohana, Chuo-Ku, Chiba, 260-8670 Japan; 2 Department of Respirology, Chibaken Saiseikai Narashino Hospital, Narashino, 275-8580 Japan; 3 Division of Cardiovascular Medicine, Department of Internal Medicine, Kobe University Graduate School of Medicine, Kobe, 650-0017 Japan; 4 Department of Cardiovascular Surgery, Eastern Chiba Medical Center, Togane, 283-8686 Japan; 5 Biostatistics Section, Clinical Research Center, Chiba University Hospital, Chiba, 260-8670 Japan

**Keywords:** Chronic thromboembolic pulmonary hypertension, Mixed venous oxygen tension, Pulmonary artery hypertension, Risk stratification, Tissue hypoxia, Respiratory care, Pulmonology

## Abstract

**Background:**

The prognostic value of mixed venous oxygen tension (PvO_2_) at pulmonary hypertension diagnosis treated with selective pulmonary vasodilators remains unclear. This study sought to investigate the association of PvO_2_ with long-term prognosis in pulmonary arterial hypertension (PAH) and medically treated chronic thromboembolic pulmonary hypertension (CTEPH) and to identify the distinct mechanisms influencing tissue hypoxia in patients with CTEPH or PAH.

**Methods:**

We retrospectively analyzed data from 138 (age: 50.2 ± 16.6 years, 81.9% women) and 268 (age: 57.4 ± 13.1 years, 72.8% women) patients with PAH and CTEPH, respectively, diagnosed at our institution from 1983 to 2018. We analyzed the survival rates of patients with/without tissue hypoxia (PvO_2_ < 35 mmHg) and identified their prognostic factors based on the pulmonary hypertension risk stratification guidelines.

**Results:**

Survival was significantly poorer in patients with tissue hypoxia than in those without it for PAH (*P* = 0.001) and CTEPH (*P* = 0.017) treated with selective pulmonary vasodilators. In patients with PAH, PvO_2_ more strongly correlated with prognosis than other hemodynamic prognostic factors regardless of selective pulmonary vasodilators usage. PvO_2_ was the only significant prognostic factor in patients with CTEPH treated with pulmonary hypertension medication. Patients with CTEPH experiencing tissue hypoxia exhibited significantly poorer survival than those in the intervention group (*P* < 0.001). PvO_2_ more strongly correlated with the cardiac index (CI) than the alveolar-arterial oxygen gradient (A-aDO_2_) in PAH; whereas in CTEPH, PvO_2_ was more strongly correlated with A-aDO_2_ than with CI.

**Conclusions:**

PvO_2_ may represent a crucial prognostic factor for pulmonary hypertension. The prognostic impact of tissue hypoxia affects different aspects of PAH and CTEPH, thereby reflecting their distinct pathogenesis.

**Supplementary Information:**

The online version contains supplementary material available at 10.1186/s12890-022-02073-0.

## Background

Pulmonary hypertension (PH) is a progressive disease characterized by abnormal remodeling of small pulmonary arteries, elevated pulmonary arterial pressure, and increased pulmonary vascular resistance (PVR) owing to various etiologies; it can lead to right ventricular dysfunction and death [1]. Currently, selective pulmonary vasodilators that act via three different pathways are available for treating pulmonary arterial hypertension (PAH), and clinicians recommend initial combination therapy [2, 3]. Despite the establishment of treatment algorithms and reduced mortality in PAH, the number of patients in the red zone (the high-risk group) as per the European Society of Cardiology (ESC) and European Respiratory Society (ERS) PH risk stratification is still high [4]. Conversely, patients with medically treated chronic thromboembolic pulmonary hypertension (CTEPH) not indicated for pulmonary endarterectomy (PEA) or balloon pulmonary angioplasty (BPA) have poor prognosis [5].

The high mortality of PAH warrants an accurate prognosis estimation for guiding its management. The 2015 ESC/ERS PH risk stratification guidelines proposed the right atrial pressure (RAP), cardiac index (CI), and mixed venous oxygen saturation (SvO_2_) as hemodynamic prognostic risk factors for PAH, and French risk stratification also defined intermediate-risk (yellow zone) or high-risk (red zone) criteria as RAP ≥ 8 mmHg and CI < 2.5 L/min/m^2^ [6, 7]. Sandqvist et al. reported that the ESC/ERS risk stratification for PAH also predicted survival in CTEPH [8]. Hurdmane et al. reported that age, SvO_2_, and World Health Organization (WHO) functional class were independent predictors of survival in 101 registered patients with PH and chronic obstructive pulmonary disease (COPD) in the ASPIRE (Assessing the Spectrum of Pulmonary Hypertension Identified at a Referral Centre) study; moreover, an SvO_2_ of 65% was reported as a better threshold for defining poor outcomes [9]. SvO_2_ improves the adequacy of tissue oxygenation, which is an essential component of normal organ function. Moreover, SvO_2_ and mixed venous oxygen tension (PvO_2_) are related to tissue oxygenation; an SvO_2_ of 65% corresponds to a PvO_2_ of 35 mmHg according to the oxygen dissociation curve in the normal state [10]. Mithoefer et al. reported that normal PvO_2_ values negatively correlate with age; at 70 years, PvO_2_ decreases to approximately the lower limit of 35 mmHg [11, 12]. Accordingly, a PvO_2_ < 35 mmHg is used as a key clinical threshold for tissue hypoxia in COPD and PH [10, 11, 13–15]. Physiologically, unlike SvO_2_, PvO_2_ reflects actual tissue hypoxia. However, the relevance of tissue hypoxia (defined by a PvO_2_ < 35 mmHg) in PAH and CTEPH pathogenesis has not been reported. Moreover, tissue oxygenation is reportedly superior to cardiac function for assessing the disease severity and predicting survival in PAH [16]. Kapitan et al. [17] reported that the main cause of hypoxemia in CTEPH was ventilation-perfusion mismatch, and that low PvO_2_, and PEA improved both; nonetheless, these issues remain controversial. Thus, in the present study, we aimed to investigate the association of PvO_2_ with long-term prognosis in patients with PAH and medically treated CTEPH and to determine the relevance of PvO_2_ relative to other prognostic factors. Furthermore, we aimed to clarify and compare the mechanisms underlying tissue hypoxia in CTEPH and PAH.

## Methods

### Study participants and design

This retrospective cohort study included patients diagnosed with PAH or CTEPH (naïve patients who had not received PH treatment) at the Chiba University Hospital between January 1983 and December 2018 (Additional file 2: Fig. S1). These patients were identified from the Chiba University Hospital Pulmonary Hypertension Center Registry. Hemodynamic parameters were measured during the first right heart catheterization (RHC). The patients were followed up until September 2021. Follow-up data were obtained by contacting the patients or their physicians.

### Ethical approval

This study was conducted in accordance with the tenets of the amended Declaration of Helsinki. Patient identity was concealed in this study, and data were compiled according to the requirements of the Japanese Ministry of Health, Labour and Welfare, which is dedicated to privacy, information technology, and civil rights. The research protocol for this study was approved by the Research Ethics Committee of the Chiba University School of Medicine (Approval No.: 2584); we had already performed "opt-out" by notifying or disclosing information. Written informed consent was obtained from all patients who were enrolled since 2009, when the requirement became mandatory (Approval No.: 826). In the case of patients who died before 2008, written informed consent was obtained from their next of kin when we examined prognosis in the relevant study (Approval No.: 84). The study database was anonymized, and all experiments were performed in accordance with the relevant guidelines and regulations.

### PAH

Patients with a mean pulmonary artery pressure (mPAP) ≥ 25 mmHg, pulmonary artery wedge pressure (PAWP) ≤ 15 mmHg, and PVR > 3 Wood units were considered to have PAH [6]. Patients suspected of complicating PH due to chronic pulmonary disease were excluded where possible by having computed tomography scans read by two respiratory experts. We diagnosed 167 patients with PAH but excluded 13 without PvO_2_ data breathing room air, 11 who died due to other diseases during follow-up, 4 with left to right shunt due to atrial septal defect, and 1 with anemia (hemoglobin ≤ 8 g/dL) (Additional file 2: Fig. S1A). Of the remaining 138 patients analyzed, 61 were diagnosed with idiopathic or hereditary PAH (Additional file 1: Table S1). By July 2021, 61 patients had died (35 patients treated with selective pulmonary vasodilators) and 77 had survived. The mean follow-up period was 7.0 ± 7.0 years.

### CTEPH

Robust evidence supports a new definition of pre-capillary PH, referred to as CTEPH [5]. Patients with CTEPH were defined as follows: (1) mPAP ≥ 25 mmHg and PAWP ≤ 15 mmHg; (2) persistent symptoms > 3 months; and (3) chronic thrombi on lung perfusion images, enhanced computed tomography, or pulmonary angiography. We diagnosed 319 patients with CTEPH but excluded 5 with accompanying respiratory diseases, 3 without data on PvO_2_ breathing room air, 19 who died due to other diseases during follow-up, and 24 who died perioperatively (Additional file 2: Fig. S1B). Five patients had hyperthyroidism and six had hypothyroidism; however, they were well managed with treatment, and hence these patients were included. No severe anemia was observed. The remaining 268 patients were classified into three groups according to the treatment strategy. Patients who underwent PEA and BPA (either BPA after PEA or PEA after BPA) were classified into the PEA/BPA group. Patients treated with selective pulmonary vasodilators composed the PH medication group. Patients treated solely with anticoagulants and oxygen therapy composed the supportive group. By July 2021, 60 patients had died (20 patients with the PEA/BPA group and 25 with the PH medication group) and 208 survived. The mean follow-up period was 9.6 ± 6.9 years.

### RHC

All patients were admitted and underwent RHC in the supine position with zero point of the transducer set at the intersection of the fourth intercostal space and mid-chest level. The pulmonary pressure was measured from the superior vena cava to PAWP at end-expiration in room air conditions whenever possible. The cardiac output was measured using a thermodilution method averaging at least three within 10% variation, and the CI and PVR were calculated.

### Blood gas analysis

Mixed venous blood for gas analysis was obtained from the distal tip of the Swan–Ganz catheter and was freely located in the major pulmonary artery. Blood gas analysis of arterial oxygen tension (PaO_2_) was performed by puncture of the radial or femoral artery. All blood gas analyses were performed in room air during the RHC and measured at the time of (1) the first diagnosis of pulmonary hypertension and (2) the latest follow-up. The alveolar-arterial oxygen gradient (A-aDO_2_) was calculated using the following equation: A-aDO_2_ = 150 − PaCO_2_/0.8 − PaO_2_, where PaCO_2_ refers to the arterial carbon dioxide tension.

### Statistical analysis

The results are expressed as mean ± standard deviation for continuous variables and as numbers and percentages for categorical variables. If the results did not show a normal distribution, a nonparametric test was performed. Comparisons between the groups were performed using the chi-squared test, Mann–Whitney U test, or analysis of variance with the Kruskal–Wallis test as appropriate. The Kaplan–Meier method was used to estimate the disease-specific and overall survival using the log-rank test for comparison. Differences between continuous variables, such as hemodynamic or oxygenation parameters, were compared using the paired *t*-test. Univariate and multivariate Cox proportional hazard models were used to examine the prognostic factors. Variable selection was based on the ESC/ERS risk stratification 2015 in addition to age, mPAP, PVR, A-aDO_2_, brain natriuretic peptide (BNP), 6-min walk distance (6MWD), percent predicted forced vital capacity, percent predicted carbon monoxide diffusing capacity (DLCO, %pred.), and WHO functional class. The predicted survival span in elderly patients is short, and mPAP decreased as patients with PAH became older [18]. A multivariate analysis was carried out with the addition of age, which was considered important as a prognostic factor, and the hemodynamic parameters, included in the 2015 ESC/ERS PH risk stratification guidelines and French risk stratification intermediate-risk (yellow zone) or high-risk (red zone) criteria. However, as ESC/ERS risk stratification for CTEPH was not widely accepted, we built another model in the PEA/BPA and PH medication groups based on significant prognostic factors in the univariate analysis. We considered a maximum of five parameters in a multivariate analysis for the number of events (range 20–35). Pearson’s correlation coefficient and multiple regression analysis were used to estimate the correlational and confounding factors for PvO_2_. Statistical significance was set at *P* < 0.05. Significant differences in the comparison of two survival curves among the three groups were determined using Bonferroni correction. All statistical analyses were performed using GraphPad Prism 8^®^ (GraphPad Software, Inc., La Jolla, CA, USA) and JMP Pro 15 (Japanese version; SAS Institute Inc., Tokyo, Japan).

## Results

### Patient characteristics stratified by PvO_2_ of 35 mmHg and categorized by treatment

The mean age of the 138 patients with PAH was 50.2 ± 16.6 years; the majority were women (81.9%), and 44.2% were diagnosed with idiopathic (IPAH) or heritable (HPAH) PAH (Additional file 1: Table S1). Table 1 shows the characteristics of patients with PAH stratified by PvO_2_ of 35 mmHg at diagnosis. Patients with PvO_2_ < 35 mmHg showed that most parameters (WHO functional class, hemodynamics, gas exchange, and even exercise endurance) were significantly worse compared to those without. Regarding the characteristics of patients treated and not treated with selective pulmonary vasodilators, patients treated with selective pulmonary vasodilators were significantly older and had significantly lower PaO_2_ and higher A-aDO_2_ than in untreated patients; however, no significant differences were observed in the other hemodynamic characteristics (Additional file 1: Table S2). In Japan, epoprostenol and bosentan have been available since 1999 and 2005, respectively. Among the untreated group, 25 patients died before 1999, 3 had oxygen therapy only for PH associated with portal hypertension, 3 had connective tissue disease (CTD) associated PAH that required intensified treatment of CTD with immunosuppressive drugs, and 3 had side effects from selective pulmonary vasodilators that failed to treat the PAH.

**Table 1:** Characteristics of patients with PAH stratified by PvO_2_ of 35 mmHg

Variable	PvO_2_		*P*
	≥ 35 mmHg	< 35 mmHg	
N	85	53	
Age (years)	48.0 ± 17.5	53.6 ± 14.6	0.066
Sex (F/M)	70/15	43/10	0.856
RAP (mmHg)	4.6 ± 3.7	6.8 ± 4.7	**0.005**
mPAP (mmHg)	42.0 ± 10.8	51.8 ± 14.1	** < 0.001**
CI (L/min/m^2^)	3.1 ± 0.7	2.2 ± 0.6	** < 0.001**
PVR (W.U)	7.8 ± 3.5	14.4 ± 7.7	** < 0.001**
PaO_2_ (mmHg)	77.2 ± 11.6	62.5 ± 12.1	** < 0.001**
PvO_2_ (mmHg)	39.0 ± 2.8	31.0 ± 2.6	** < 0.001**
SvO_2_ (mmHg)	72.0 ± 5.2	58.5 ± 7.1	** < 0.001**
A-aDO_2_ (mmHg)	26.9 ± 11.9	43.4 ± 13.1	** < 0.001**
BNP (pg/mL)	128.0 ± 235.5	460.0 ± 523.0	** < 0.001**
6MWD (m)	395.6 ± 106.3	306.8 ± 100.0	** < 0.001**
FVC, %pred. (%)	87.2 ± 16.8	81.4 ± 22.5	0.140
DLCO, %pred. (%)	61.8 ± 20.1	50.9 ± 22.9	**0.011**
WHO functional class I, II, III, IV	6/44/34/1	0/16/29/8	** < 0.001**
Medical treatment	63 (74.1%)	41 (77.4%)	0.667
Combination pulmonary vasodilators, n (%)	36 (42.4%)	21 (39.6%)	0.751
ERA, n (%)	44 (51.8%)	23 (43.4%)	0.339
PDE5-I, n (%)	38 (44.7%)	23 (43.4%)	0.880
Prostacyclin, n (%)	38 (44.7%)	29 (54.7%)	0.252
sGCS, n (%)	5 (5.9%)	2 (3.8%)	0.583

Statistically significant,* P* < 0.05, are shown in bold

Data are presented as mean ± standard deviation or numbers

*A-aDO*_*2*_ alveolar-arterial oxygen gradient, *BNP* brain natriuretic peptide, *CI* cardiac index *DLCO, %pred.* percent predicted carbon monoxide diffusing capacity, *ERA* endothelin receptor antagonists, *FVC, %pred.* percent predicted forced vital capacity, *mPAP* mean pulmonary arterial pressure, *PAH* pulmonary arterial hypertension, *PaO*_*2*_ arterial oxygen tension, *PDE5-I* phosphodiesterase type 5 inhibitors, *PvO*_*2*_ mixed venous oxygen tension, *PVR* pulmonary vascular resistance, *RAP* right arterial pressure, *sGCS* soluble guanylate cyclase stimulator, *SvO*_*2*_ mixed venous oxygen saturation, *WHO* World Health Organization, *W.U* Wood units, *6MWD* 6-min walk distance

The mean age of the 268 patients with CTEPH was 57.4 ± 13.1 years, and the majority were women (72.8%). Table 2 indicates the characteristics of patients with CTEPH stratified by PvO_2_ of 35 mmHg at diagnosis. The results were similar to those in PAH: patients with PvO_2_ < 35 mmHg showed that WHO functional class, hemodynamics, gas exchange, and even exercise endurance were significantly worse compared to those without in CTEPH. There was a significant difference in treatment between the two groups. Additional file 1: Table S3 summarizes the patient characteristics according to the treatment modality. The PEA/BPA group was significantly younger and had a significantly higher mPAP than the PH medication group. In the PEA/BPA group, 51 patients had residual PH and were treated with selective pulmonary vasodilators.

**Table 2:** Characteristics of patients with CTEPH stratified by PvO_2_ of 35 mmHg

Variable	PvO_2_		*P*
	≥ 35 mmHg	< 35 mmHg	
N	84	184	
Age (years)	55.6 ± 14.7	58.2 ± 12.1	0.297
Sex (F/M)	57/27	138/46	0.223
RAP (mmHg)	3.3 ± 2.6	6.3 ± 4.1	** < 0.001**
mPAP (mmHg)	37.3 ± 8.8	47.1 ± 10.3	** < 0.001**
CI (L/min/m^2^)	3.0 ± 0.6	2.4 ± 0.6	** < 0.001**
PVR (W.U)	6.6 ± 2.8	10.9 ± 4.2	** < 0.001**
PaO_2_ (mmHg)	66.2 ± 9.5	55.0 ± 8.0	** < 0.001**
PvO_2_ (mmHg)	37.6 ± 2.4	31.1 ± 2.7	** < 0.001**
SvO_2_ (mmHg)	70.1 ± 4.0	58.9 ± 6.0	** < 0.001**
A-aDO_2_ (mmHg)	36.0 ± 11.0	49.2 ± 8.7	** < 0.001**
BNP (pg/mL)	69.2 ± 109.3	285.0 ± 329.2	** < 0.001**
6MWD (m)	409.6 ± 99.9	340.3 ± 92.2	** < 0.001**
FVC, %pred. (%)	97.9 ± 20.4	93.1 ± 18.0	**0.031**
DLCO, %pred. (%)	79.1 ± 18.8	73.3 ± 21.1	**0.021**
WHO functional class I, II, III, IV	4/45/33/2	1/49/121/13	** < 0.001**
PEA/BPA, n (%)	49 (58.3%)	128 (69.6%)	0.005*
PH medication, n (%)	20 (23.8%)	46 (25.0%)	
Supportive, n (%)	15 (17.9%)	10 (5.4%)	

Statistically significant,* P* < 0.05, are shown in bold

Data are presented as mean ± standard deviation or numbers

*A-aDO*_*2*_ alveolar-arterial oxygen gradient, *BNP* brain natriuretic peptide, *BPA* balloon pulmonary angioplasty, *CI* cardiac index, *CTEPH* chronic thromboembolic pulmonary hypertension, *DLCO, %pred.* percent predicted carbon monoxide diffusing capacity, *FVC, %pred.* percent predicted forced vital capacity, *mPAP* mean pulmonary arterial pressure, *PaO*_*2*_ arterial oxygen tension, *PEA* pulmonary endarterectomy, *PH* pulmonary hypertension, *PvO*_*2*_ mixed venous oxygen tension, *PVR* pulmonary vascular resistance, *RAP* right arterial pressure, *SvO*_*2*_ mixed venous oxygen saturation, *WHO* World Health Organization, *W.U* Wood units, *6MWD* 6-min walk distance

*There was a significant difference in treatment between the two groups

### Survival analysis of the treatment groups

**Fig. 1:**
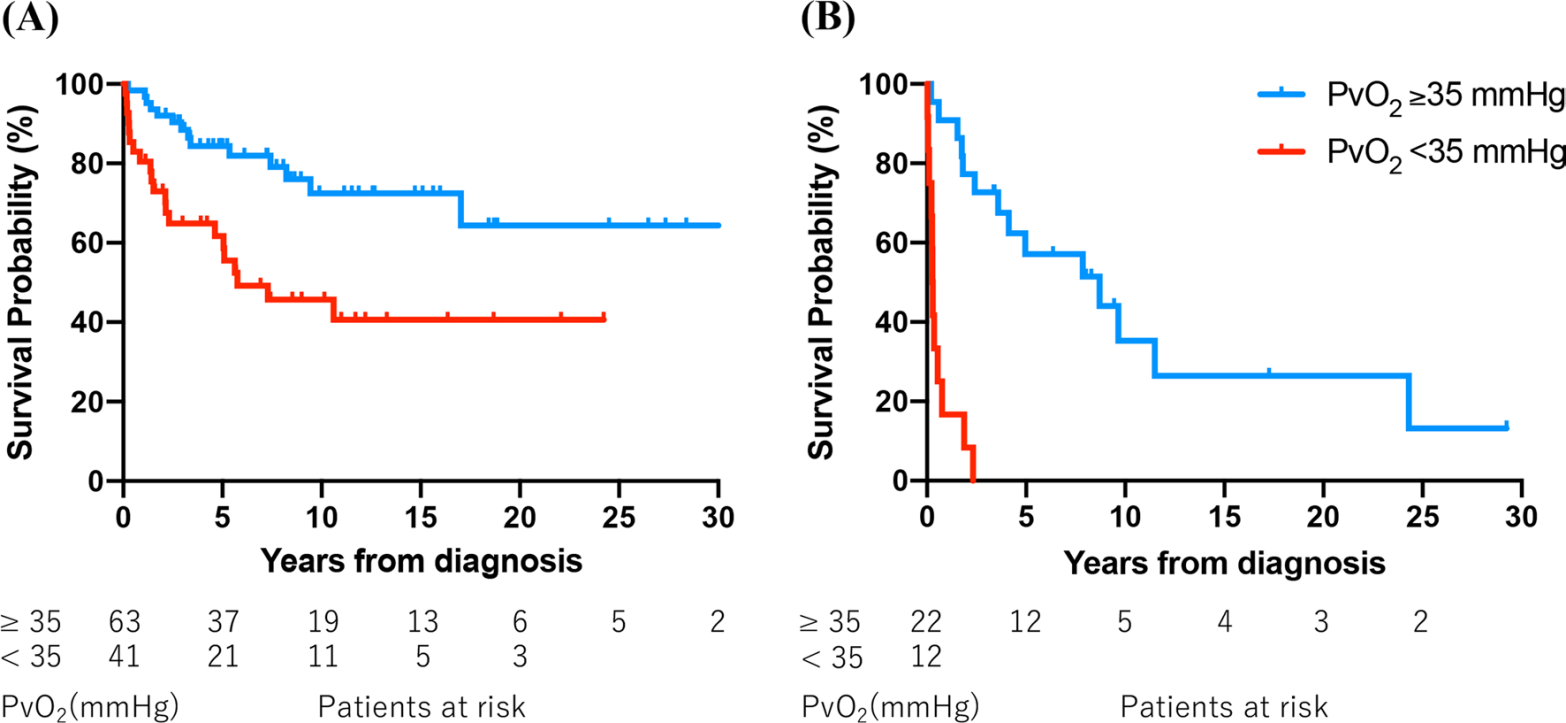
Kaplan–Meier survival curves stratified by the presence of tissue hypoxia (PvO_2_ < 35 mmHg) in patients with PAH. **A** Group treated with selective pulmonary vasodilators (*P* = 0.001). **B** Untreated group (*P* < 0.001). *PAH* pulmonary arterial hypertension, *PvO*_*2*_ mixed venous oxygen tension

**Fig. 2:**
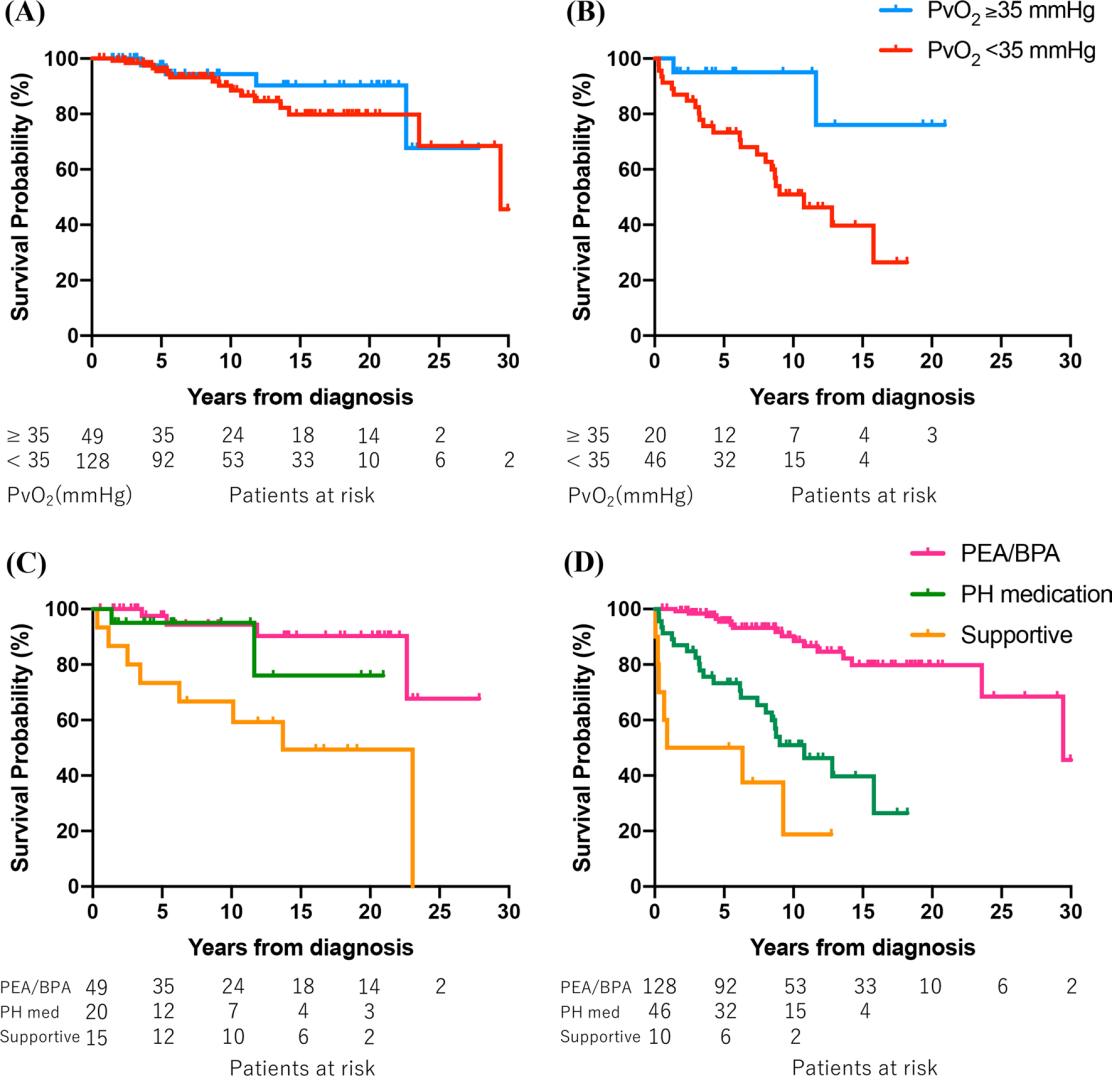
**A**, **B** Kaplan–Meier survival curves stratified by the presence of tissue hypoxia (PvO_2_ < 35 mmHg) in patients with CTEPH. **A** PEA/BPA group (*P* = 0.445). **B** PH medication group (*P* = 0.017). **C** Comparison among the PEA/BPA, PH medication, and supportive groups in the absence of tissue hypoxia (*P* = 0.002). There is no significant difference in survival between the PEA/BPA and PH medication groups (*P* = 0.366). **D** Comparison among the three groups in the presence of tissue hypoxia (*P* < 0.001). The PEA/BPA group exhibits better survival than the PH medication group (*P* < 0.001). *BPA* balloon pulmonary angioplasty, *CTEPH* chronic thromboembolic pulmonary hypertension, *PEA* pulmonary endarterectomy, *PH* pulmonary hypertension, *PvO*_*2*_ mixed venous oxygen tension

Patients with PAH and tissue hypoxia at diagnosis had significantly poorer survival than those without tissue hypoxia, regardless of treatment with selective pulmonary vasodilators (treated: *P* = 0.001, Fig. 1A; untreated: *P* < 0.001, Fig. 1B). These results were similar in the IPAH/HPAH group (treated: *P* = 0.006, Additional file 3: Fig. S2A; untreated: *P* = 0.011, Additional file 3: Fig. S2B). For patients with CTEPH in the PEA/BPA group, there was no significant difference in survival between those with and without tissue hypoxia (*P* = 0.445, Fig. 2A). However, survival was significantly poorer in patients with tissue hypoxia than in those without tissue hypoxia in the PH medication (*P* = 0.017, Fig. 2B) and supportive (*P* = 0.043) groups. In the absence of tissue hypoxia at diagnosis, there was a significant difference in survival among the three groups, with poor prognosis in the supportive group (*P* = 0.002); however, no significant difference was observed between the PEA/BPA and PH medication groups (*P* = 0.366, Fig. 2C). In the presence of tissue hypoxia at diagnosis, significant differences in survival were observed among the three groups (*P* < 0.001) and between the PEA/BPA and PH medication groups (*P* < 0.001, Fig. 2D).

### Prognostic factors stratified by treatment

Univariate analyses revealed that age, mPAP, CI, CI < 2.5 L/min/m^2^, PVR, PaO_2_, PvO_2_, PvO_2_ < 35 mmHg, SvO_2_, A-aDO_2_, BNP, 6MWD, DLCO, %pred., WHO functional class, and medication significantly correlated with prognosis in all PAH patients. Multivariate analyses were made by two models: using continuous variables by ESC/ERS risk stratification, medication, and age as model 1; and using categorical variables by ESC/ERS and French risk stratification in yellow and red zone, age, and medication as model 2. In model 1, age, CI, PvO_2_, and medication were significant prognostic factors, while in model 2, PvO_2_ < 35 mmHg, CI < 2.5 L/min/m^2^, age, and medication were significant prognostic factors (Table 3).

**Table 3:** Univariate and multivariate analyses of prognostic factors for patients with PAH (N = 138)

Variable	Univariate		Multivariate model 1		Multivariate model 2	
	HR (95% CIv)	*P*	HR (95% CIv)	*P*	HR (95% CIv)	*P*
Age (years)	1.03 (1.012–1.047)	** < 0.001**	1.04 (1.018–1.057)	** < 0.001**	1.03 (1.012–1.050)	** < 0.001**
RAP (mmHg)	0.99 (0.928–1.058)	0.822	0.95 (0.877–1.015)	0.124		
mPAP (mmHg)	1.03 (1.008–1.048)	**0.008**				
CI (L/min/m^2^)	0.23 (0.142–0.367)	** < 0.001**	0.42 (0.244–0.706)	** < 0.001**		
PVR (W.U)	1.15 (1.110–1.196)	** < 0.001**				
PaO_2_ (mmHg)	0.98 (0.958–0.997)	**0.026**				
PvO_2_ (mmHg)	0.87 (0.816–0.918)	** < 0.001**	0.86 (0.784–0.931)	** < 0.001**		
PvO_2_ < 35 mmHg	2.86 (1.719–4.774)	** < 0.001**			3.13 (1.707–5.735)	** < 0.001**
RAP ≥ 8 mmHg	0.60 (0.312–1.156)	0.108			0.58 (0.288–1.178)	0.132
CI < 2.5 L/min/m^2^	3.65 (2.151–6.183)	** < 0.001**			2.87 (1.599–5.146)	** < 0.001**
SvO_2_ (mmHg)	0.94 (0.918–0.964)	** < 0.001**				
A-aDO_2_ (mmHg)	1.03 (1.013–1.052)	**0.001**				
BNP (pg/mL)	1.00 (1.001–1.002)	** < 0.001**				
6MWD (m)	0.99 (0.990–0.997)	**0.001**				
FVC, %pred. (%)	0.99 (0.970–1.003)	0.111				
DLCO, %pred. (%)	0.98 (0.964–0.996)	**0.017**				
WHO functional class I + II (vs. III + IV)	0.31 (0.176–0.534)	** < 0.001**				
Medication	0.33 (0.196–0.543)	** < 0.001**	0.15 (0.084–0.285)	** < 0.001**	0.18 (0.100–0.321)	** < 0.001**

Statistically significant,* P* < 0.05, are shown in bold

*A-aDO*_*2*_ alveolar-arterial oxygen gradient, *BNP* brain natriuretic peptide, *CI* cardiac index, *CIv* confidence interval, *DLCO, %pred.* percent predicted carbon monoxide diffusing capacity, *FVC, %pred.* percent predicted forced vital capacity, *HR* hazard ratio, *mPAP* mean pulmonary arterial pressure, *PAH* pulmonary arterial hypertension, *PaO*_*2*_ arterial oxygen tension, *PvO*_*2*_ mixed venous oxygen tension, *PVR* pulmonary vascular resistance, *RAP* right arterial pressure, *SvO*_*2*_ mixed venous oxygen saturation, *WHO* World Health Organization, *W.U* Wood units, *6MWD* 6-min walk distance

Model 1: Multivariate analysis by ESC/ERS risk stratification, medication and age

Model 2: Multivariate analysis by ESC/ERS and French risk stratification in yellow and red zone, medication and age

Furthermore, additional analyses with/without treatment in PAH showed that age, CI, CI < 2.5 L/min/m^2^, PVR, PaO_2_, PvO_2_, PvO_2_ < 35 mmHg, SvO_2_, A-aDO_2_, BNP, 6MWD, and WHO functional class were significant prognostic factors in the group treated with selective pulmonary vasodilators (Table 4), and that mPAP, CI, CI < 2.5 L/min/m^2^, PVR, PvO_2_, PvO_2_ < 35 mmHg, SvO_2_, A-aDO_2_, DLCO, %pred., and WHO functional class were significantly correlated with prognosis in the untreated group (Table 5). As to multivariate analyses, in the group treated with selective vasodilators, PvO_2_, CI, and age were prognostic factors in both models 1 and 2 (Table 4). Whereas in the untreated group, PvO_2_ was the only significant prognostic factor in models 1 and 2 (Table 5).

**Table 4:** Univariate and multivariate analyses of prognostic factors for patients with PAH with pulmonary vasodilator treatment

Variable	Univariate		Multivariate model 1		Multivariate model 2	
	HR (95% CIv)	*P*	HR (95% CIv)	*P*	HR (95% CIv)	*P*
Age (years)	1.05 (1.029–1.084)	** < 0.001**	1.06 (1.029–1.090)	** < 0.001**	1.05 (1.021–1.078)	** < 0.001**
RAP (mmHg)	1.00 (0.918–1.082)	0.990	0.94 (0.856–1.017)	0.120		
mPAP (mmHg)	1.02 (0.987–1.048)	0.270				
CI (L/min/m^2^)	0.23 (0.110–0.440)	** < 0.001**	0.42 (0.170–0.949)	**0.037**		
PVR (W.U)	1.15 (1.078–1.220)	** < 0.001**				
PaO_2_ (mmHg)	0.96 (0.930–0.985)	**0.002**				
PvO_2_ (mmHg)	0.84 (0.776–0.914)	** < 0.001**	0.84 (0.740–0.937)	**0.002**		
PvO_2_ < 35 mmHg	2.89 (1.466–5.684)	**0.002**			2.36 (1.173–4.736)	**0.015**
RAP ≥ 8 mmHg	0.59 (0.257–1.349)	0.188			0.55 (0.225–1.371)	0.185
CI < 2.5 L/min/m^2^	3.25 (1.633–6.453)	** < 0.001**			2.60 (1.278–5.276)	**0.007**
SvO_2_ (mmHg)	0.93 (0.908–0.966)	** < 0.001**				
A-aDO_2_ (mmHg)	1.04 (1.016–1.068)	**0.001**				
BNP (pg/mL)	1.00 (1.001–1.002)	** < 0.001**				
6MWD (m)	0.99 (0.990–0.998)	**0.002**				
FVC, %pred. (%)	0.98 (0.965–1.003)	0.105				
DLCO, %pred. (%)	0.99 (0.969–1.007)	0.211				
WHO functional class I + II (vs. III + IV)	0.39 (0.195–0.772)	**0.006**				

Statistically significant,* P* < 0.05, are shown in bold

*A-aDO*_*2*_ alveolar-arterial oxygen gradient, *BNP* brain natriuretic peptide, *CI* cardiac index, *CIv* confidence interval, *DLCO, %pred.* percent predicted carbon monoxide diffusing capacity, *FVC, %pred.* percent predicted forced vital capacity, *HR* hazard ratio, *mPAP* mean pulmonary arterial pressure, *PAH* pulmonary arterial hypertension, *PaO*_*2*_ arterial oxygen tension, *PvO*_*2*_ mixed venous oxygen tension, *PVR* pulmonary vascular resistance, *RAP* right arterial pressure, *SvO*_*2*_ mixed venous oxygen saturation, *WHO* World Health Organization, *W.U* Wood units, *6MWD* 6-min walk distance

Model 1: Multivariate analysis by ESC/ERS risk stratification and age

Model 2: Multivariate analysis by ESC/ERS and French risk stratification in yellow and red zone, and age

**Table 5:** Univariate and multivariate analyses of prognostic factors for patients with PAH without pulmonary vasodilator treatment

Variable	Univariate		Multivariate model 1		Multivariate model 2	
	HR (95% CIv)	*P*	HR (95% CIv)	*P*	HR (95% CIv)	*P*
Age (years)	1.02 (0.993–1.044)	0.155	1.02 (0.987–1.045)	0.279	1.02 (0.987–1.046)	0.272
RAP (mmHg)	1.06 (0.924–1.220)	0.416	0.95 (0.797–1.195)	0.533		
mPAP (mmHg)	1.03 (1.001–1.050)	**0.041**				
CI (L/min/m^2^)	0.31 (0.170–0.531)	** < 0.001**	0.56 (0.255–1.144)	0.112		
PVR (W.U)	1.12 (1.066–1.187)	** < 0.001**				
PaO_2_ (mmHg)	0.98 (0.946–1.010)	0.170				
PvO_2_ (mmHg)	0.77 (0.690–0.854)	** < 0.001**	0.82 (0.700–0.937)	**0.003**		
PvO_2_ < 35 mmHg	11.94 (4.007–35.554)	** < 0.001**			6.45 (1.626–25.568)	**0.003**
RAP ≥ 8 mmHg	0.98 (0.333–2.891)	0.972			0.84 (0.263–2.658)	0.759
CI < 2.5 L/min/m^2^	6.18 (2.551–14.950)	** < 0.001**			2.57 (0.771–8.548)	0.135
SvO_2_ (mmHg)	0.86 (0.812–0.916)	** < 0.001**				
A-aDO_2_ (mmHg)	1.05 (1.012–1.085)	**0.009**				
BNP (pg/mL)	1.00 (0.992–1.007)	0.903				
6MWD (m)	0.99 (0.952–1.009)	0.105				
FVC, %pred. (%)	0.99 (0.965–1.026)	0.719				
DLCO, %pred. (%)	0.96 (0.917–0.991)	**0.011**				
WHO functional class I + II (vs. III + IV)	0.16 (0.047–0.572)	** < 0.001**				

Statistically significant,* P* < 0.05, are shown in bold

*A-aDO*_*2*_ alveolar-arterial oxygen gradient, *BNP* brain natriuretic peptide, *CI* cardiac index, *CIv* confidence interval, *DLCO, %pred.* percent predicted carbon monoxide diffusing capacity, *FVC, %pred.* percent predicted forced vital capacity, *HR* hazard ratio, *mPAP* mean pulmonary arterial pressure, *PAH* pulmonary arterial hypertension, *PaO*_*2*_ arterial oxygen tension, *PvO*_*2*_ mixed venous oxygen tension, *PVR* pulmonary vascular resistance, *RAP* right arterial pressure, *SvO*_*2*_ mixed venous oxygen saturation, *WHO* World Health Organization, *W.U* Wood units, *6MWD* 6-min walk distance

Model 1: Multivariate analysis by ESC/ERS risk stratification and age

Model 2: Multivariate analysis by ESC/ERS and French risk stratification in yellow and red zone, and age

In all patients with CTEPH, PvO_2_ and PEA/BPA treatment were prognostic factors, however, PvO_2_ < 35 mmHg was not (Table 6). Multivariate analyses showed that PEA/BPA treatment and PvO_2_ or PvO_2_ < 35 mmHg were significant prognostic factors by models 1 and 2, respectively (Table 6).

**Table 6:** Univariate and multivariate analyses of prognostic factors for patients with CTEPH (N = 277)

Variable	Univariate		Multivariate model 1		Multivariate model 2	
	HR (95% CIv)	*P*	HR (95% CIv)	*P*	HR (95% CIv)	*P*
Age (years)	1.02 (0.995–1.041)	0.139	1.01 (0.987–1.032)	0.439	1.01 (0.986–1.030)	0.461
RAP (mmHg)	1.07 (1.009–1.137)	**0.026**	1.03 (0.952–1.117)	0.425		
mPAP (mmHg)	1.02 (0.994–1.043)	0.136				
CI (L/min/m^2^)	0.66 (0.426–0.996)	**0.048**	0.84 (0.521–1.353)	0.479		
PVR (W.U)	1.11 (1.052–1.178)	** < 0.001**				
PaO_2_ (mmHg)	0.98 (0.955–1.011)	0.241				
PvO_2_ (mmHg)	0.91 (0.848–0.976)	**0.008**	0.89 (0.815–0.969)	**0.007**		
PvO_2_ < 35 mmHg	1.59 (0.873–2.913)	0.115			2.46 (1.140–5.307)	**0.019**
RAP ≥ 8 mmHg	2.13 (1.221–3.710)	**0.011**			1.69 (0.872–3.261)	0.126
CI < 2.5 L/min/m^2^	1.58 (0.944–2.639)	0.080			1.01 (0.554–1.855)	0.966
SvO_2_ (mmHg)	0.94 (0.909–0.977)	**0.001**				
A-aDO_2_ (mmHg)	1.01 (0.986–1.035)	0.418				
BNP (pg/mL)	1.00 (1.000–1.001)	0.056				
6MWD (m)	0.99 (0.990–0.996)	** < 0.001**				
FVC, %pred. (%)	0.99 (0.969–0.996)	**0.011**				
DLCO, %pred. (%)	0.99 (0.977–1.003)	0.124				
WHO functional class I + II (vs. III + IV)	0.41 (0.206–0.808)	**0.005**				
Treatment						
PEA/BPA (vs. PH medication)	0.20 (0.107–0.367)	** < 0.001**	0.19 (0.099–0.354)	** < 0.001**	0.20 (0.108–0.385)	** < 0.001**
PEA/BPA (vs. supportive)	0.14 (0.072–0.283)	** < 0.001**	0.09 (0.045–0.187)	** < 0.001**	0.10 (0.044–0.206)	** < 0.001**
PH medication (vs. supportive)	0.72 (0.376–1.377)	0.320	0.49 (0.249–0.959)	**0.038**	0.47 (0.225–0.961)	**0.039**

Statistically significant,* P* < 0.05, are shown in bold

*A-aDO*_*2*_ alveolar-arterial oxygen gradient, *BNP* brain natriuretic peptide, *BPA* balloon pulmonary angioplasty, *CI* cardiac index, *CIv* confidence interval, *CTEPH* chronic thromboembolic pulmonary hypertension, *DLCO, %pred.* percent predicted carbon monoxide diffusing capacity, *FVC, %pred.* percent predicted forced vital capacity, *HR* hazard ratio, *mPAP* mean pulmonary arterial pressure, *PaO*_*2*_ arterial oxygen tension, *PEA* pulmonary endarterectomy, *PH* pulmonary hypertension, *PvO*_*2*_ mixed venous oxygen tension, *PVR* pulmonary vascular resistance, *RAP* right arterial pressure, *SvO*_*2*_ mixed venous oxygen saturation, *WHO* World Health Organization, *W.U* Wood units, *6MWD* 6-min walk distance

Model 1: Multivariate analysis by ESC/ERS risk stratification, treatment and age

Model 2: Multivariate analysis by ESC/ERS and French risk stratification in yellow and red zone, treatment and age

Furthermore, we conducted additional analyses by treatment modality in CTEPH. In the PEA/BPA group, only 6MWD and DLCO, %pred. correlated with the prognosis; however, in the PH medication group, RAP, mPAP, PVR, PaO_2_, PvO_2_, PvO_2_ < 35 mmHg, RAP ≥ 8 mmHg, SvO_2_, A-aDO_2_, BNP, 6MWD, and WHO functional class significantly correlated with the prognosis (Tables 7, 8). In the PEA/BPA group, multivariate analyses showed that no significant prognostic factors other than age remained in any of the models (Table 7), whereas in the PH medication group, PvO_2_ or PvO_2_ < 35 mmHg were significant prognostic factor by models 1, 2 and 3, respectively (Table 8).

**Table 7:** Univariate and multivariate analyses of prognostic factors for patients in the CTEPH PEA/BPA group

Variable	Univariate		Multivariate model 1		Multivariate model 2		Multivariate model 3	
	HR (95% CIv)	*P*	HR (95% CIv)	*P*	HR (95% CIv)	*P*	HR (95% CIv)	*P*
Age (years)	1.04 (0.991–1.085)	0.120	1.05 (1.001–1.097)	**0.044**	1.04 (0.991–1.087)	0.120	1.06 (0.999–1.139)	0.053
RAP (mmHg)	1.05 (0.929–1.158)	0.432	1.10 (0.967–1.261)	0.209				
mPAP (mmHg)	1.03 (0.987–1.083)	0.153						
CI (L/min/m^2^)	0.75 (0.334–1.560)	0.455	0.70 (0.272–1.587)	0.413				
PVR (W.U)	1.08 (0.958–1.198)	0.209						
PaO_2_ (mmHg)	1.00 (0.954–1.054)	0.873						
PvO_2_ (mmHg)	1.05 (0.928–1.197)	0.419	1.15 (0.989–1.352)	0.069			1.02 (0.831–1.267)	0.851
PvO_2_ < 35 mmHg	1.54 (0.507–4.659)	0.430			1.22 (0.368–4.039)	0.742		
RAP ≥ 8 mmHg	1.38 (0.447–4.229)	0.589			1.51 (0.438–5.215)	0.523		
CI < 2.5 L/min/m^2^	1.26 (0.515–3.064)	0.616			1.07 (0.389–2.923)	0.901		
SvO_2_ (mmHg)	0.99 (0.925–1.058)	0.730						
A-aDO_2_ (mmHg)	0.97 (0.932–1.018)	0.241						
BNP (pg/mL)	1.00 (0.998–1.002)	0.603						
6MWD (m)	0.99 (0.988–0.999)	**0.030**					0.99 (0.988–1.001)	0.098
FVC, %pred. (%)	0.96 (0.957–1.006)	0.123						
DLCO, %pred. (%)	0.98 (0.952–0.998)	**0.033**					0.98 (0.951–1.014)	0.279
WHO functional class I + II (vs. III + IV)	0.31 (0.070–1.332)	0.067						

Statistically significant,* P* < 0.05, are shown in bold

*A-aDO*_*2*_ alveolar-arterial oxygen gradient, *BNP* brain natriuretic peptide, *BPA* balloon pulmonary angioplasty, *CI* cardiac index, *CIv* confidence interval, *CTEPH* chronic thromboembolic pulmonary hypertension, *DLCO, %pred.* percent predicted carbon monoxide diffusing capacity, *FVC, %pred.* percent predicted forced vital capacity, *HR* hazard ratio, *mPAP* mean pulmonary arterial pressure, *PaO*_*2*_ arterial oxygen tension, *PEA* pulmonary endarterectomy, *PvO*_*2*_ mixed venous oxygen tension, *PVR* pulmonary vascular resistance, *RAP* right arterial pressure, *SvO*_*2*_ mixed venous oxygen saturation, *WHO* World Health Organization, *W.U* Wood units, *6MWD* 6-min walk distance

Model 1: Multivariate analysis by ESC/ERS risk stratification and age

Model 2: Multivariate analysis by ESC/ERS and French risk stratification in yellow and red zone, and age

Model 3: Multivariate analysis adding age and PvO_2_ to variables that were significant in univariate analysis

**Table 8:** Univariate and multivariate analyses of prognostic factors for patients in the CTEPH PH medication group

Variable	Univariate		Multivariate model 1		Multivariate model 2		Multivariate model 3	
	HR (95% CIv)	*P*	HR (95% CIv)	*P*	HR (95% CIv)	*P*	HR (95% CIv)	*P*
Age (years)	0.99 (0.959–1.020)	0.427	0.99 (0.951–1.025)	0.479	0.99 (0.966–1.027)	0.701	0.99 (0.955–1.033)	0.730
RAP (mmHg)	1.13 (1.034–1.237)	**0.008**	1.00 (0.864–1.127)	0.958			0.99 (0.861–1.119)	0.889
mPAP (mmHg)	1.05 (1.013–1.085)	**0.008**					0.99 (0.923–1.054)	0.688
CI (L/min/m^2^)	0.59 (0.352–1.034)	0.064	1.26 (0.569–2.983)	0.575				
PVR (W.U)	1.17 (1.081–1.264)	** < 0.001**					1.06 (0.895–1.250)	0.499
PaO_2_ (mmHg)	0.95 (0.904–0.994)	**0.025**						
PvO_2_ (mmHg)	0.74 (0.651–0.842)	** < 0.001**	0.73 (0.625–1.367)	** < 0.001**			0.77 (0.633–0.930)	**0.006**
PvO_2_ < 35 mmHg	4.83 (1.133–20.608)	**0.008**			4.26 (0.871–20.820)	**0.047**		
RAP ≥ 8 mmHg	2.80 (1.226–6.418)	**0.019**			2.08 (0.786–5.490)	0.142		
CI < 2.5 L/min/m^2^	2.11 (0.931–4.782)	0.067			0.88 (0.317–2.416)	0.798		
SvO_2_ (mmHg)	0.87 (0.816–0.924)	** < 0.001**						
A-aDO_2_ (mmHg)	1.07 (1.025–1.111)	**0.002**						
BNP (pg/mL)	1.00 (1.001–1.004)	**0.002**						
6MWD (m)	0.99 (0.984–0.994)	** < 0.001**						
FVC, %pred. (%)	0.99 (0.971–1.004)	0.112						
DLCO, %pred. (%)	0.99 (0.977–1.011)	0.492						
WHO functional class I + II (vs. III + IV)	0.22 (0.053–0.968)	**0.014**						

Statistically significant,* P* < 0.05, are shown in bold

*A-aDO*_*2*_ alveolar-arterial oxygen gradient, *BNP* brain natriuretic peptide, *CI* cardiac index, *CIv* confidence interval, *CTEPH* chronic thromboembolic pulmonary hypertension, *DLCO, %pred.* percent predicted carbon monoxide diffusing capacity, *FVC, %pred.* percent predicted forced vital capacity, *HR* hazard ratio, *mPAP* mean pulmonary arterial pressure, *PaO*_*2*_ arterial oxygen tension, *PH* pulmonary hypertension, *PvO*_*2*_ mixed venous oxygen tension, *PVR* pulmonary vascular resistance, *RAP* right arterial pressure, *SvO*_*2*_ mixed venous oxygen saturation, *WHO* World Health Organization, *W.U* Wood units, *6MWD* 6-min walk distance

Model 1: Multivariate analysis by ESC/ERS risk stratification and age

Model 2: Multivariate analysis by ESC/ERS and French risk stratification in yellow and red zone, and age

Model 3: Multivariate analysis adding age and PvO_2_ to variables that were significant in univariate analysis

### Relationships between PvO2 and CI/A-aDO2

In patients with PAH, PvO_2_ significantly correlated with CI and A-aDO_2_ (CI: r = 0.642, *P* < 0.001; A-aDO_2_: r =  − 0.549, *P* < 0.001; Additional file 4: Fig. S3A). The standardized coefficients of CI were larger than those of A-aDO_2_ in the multiple regression analysis, suggesting that CI was a more important determinant of PvO_2_ than was A-aDO_2_ (CI: β = 0.522, A-aDO_2_: β =  − 0.435; Additional file 1: Table S4).

In patients with CTEPH, PvO_2_ correlated with A-aDO_2_ and CI (CI: r = 0.470, *P* < 0.001; A-aDO_2_: r =  − 0.678, *P* < 0.001; Additional file 4: Fig. S3B). Conversely, the standardized coefficients of A-aDO_2_ were larger than that of CI, suggesting that A-aDO_2_ was a more important determinant of PvO_2_ than CI (CI: β = 0.418, A-aDO_2_: β =  − 0.645; Additional file 1: Table S5).

### Treatment-induced improvements in hemodynamics/oxygenation

We examined the post-treatment hemodynamic and oxygenation parameters at the most recent RHC (7.2 ± 7.2 years after PAH diagnosis and treatment with selective pulmonary vasodilators; 2.7 ± 4.0 years for the PEA/BPA group; and 4.8 ± 4.5 years for patients with CTEPH who received PH medication). Only mPAP and PVR were significantly improved in the PAH and PH medication groups comprising patients with CTEPH. However, no improvements were observed in oxygenation parameters, including PvO_2_ (Additional file 1: Tables S6, S7). Similar trends were observed in the IPAH/HPAH group (data not shown). In the PEA/BPA group comprising patients with CTEPH, all hemodynamic and oxygenation parameters, including PvO_2_, were significantly improved (Additional file 1: Table S7).

### Prognostic differences by eras of diagnosis in PAH and CTEPH

Recently, survival in PAH has improved significantly as upfront combination therapy has become the mainstream treatment based on data from 2008 to 2013 [19], and riociguat for CTEPH became available after 2014. Hence, we analyzed 35 patients in the PAH treated group and 14 patients in the CTEPH PH medication group diagnosed after 2014. Survival was significantly poorer in patients with tissue hypoxia at diagnosis than in those without tissue hypoxia in PAH (*P* = 0.002), and PvO_2_ significantly correlated with the prognosis in univariate analysis (*P* = 0.024). No statistical significance was seen due to the small events in multivariate analysis. No deaths were recorded among patients with CTEPH, and hence we could not perform any analyses.

## Discussion

This is a novel study to demonstrate that among the pulmonary hemodynamic parameters included in the 2015 ESC/ERS risk stratification criteria and French risk stratification criteria, lower PvO_2_ (especially PvO_2_ < 35 mmHg associated with tissue hypoxia) was a significant prognostic factor in patients with PAH and CTEPH.

Lower PvO_2_ was significantly associated with poor prognosis in patients with PAH and CTEPH independent of treatment with selective pulmonary vasodilators. However, no hemodynamic parameter (RAP, CI, and PvO_2_) correlated with the prognosis in the PEA/BPA group (Table 7). In patients with PAH and CTEPH, pulmonary vasodilator treatment improved the mPAP and PVR, but not PaO_2_ and PvO_2_, whereas invasive treatment with PEA and BPA improved both PaO_2_ and PvO_2_. Selective pulmonary vasodilators inhibit vasoconstriction, thereby decreasing the PVR and mPAP; concurrently, these agents cause a worsening in ventilation-perfusion matching, resulting in decreased PaO_2_ and maintenance of PvO_2_ in PH due to respiratory diseases [20]. Contrarily, in PAH hypocapnia is reported to be a risk of mortality, and may reflect the extent of the pulmonary vascular disease, cardiac dysfunction, and impairment in oxygen delivery [21]. Then pulmonary vasodilators may adjust hyperventilation due to pulmonary vascular disease, resulting in increased PaCO_2_. In our study, PaCO_2_ increased without worsening of A-aDO_2_ in patients with PAH and CTEPH who were treated by selective pulmonary vasodilators. Although 48% of PAH patients had worsening of A-aDO_2_ after treatment, the remaining patients demonstrated improved A-aDO_2_ with significant improvement in PVR compared to those with worsened A-aDO_2_ (ΔPVR − 3.3 ± 4.1 Wood units in the improved A-aDO_2_ group versus − 1.0 ± 4.5 Wood units in the worsened A-aDO_2_ group, *P* = 0.010) (data not shown). Thus, long-term effects of selective pulmonary vasodilators on ventilation-perfusion mismatch may not be significant in PAH. However, PvO_2_ remained a strong prognostic factor even in patients who received selective pulmonary vasodilators. It may be caused by a multi-factorial mechanism related to worsening of PaO_2_ as well as change in PVR and cardiac output. Conversely, PEA and BPA treatment was more effective in improving hemodynamics, as well as PaO_2_ and PvO_2_. These data are consistent with those reported in previous studies by Tanabe et al. [22] and Isobe et al. [23] suggesting that baseline PvO_2_ is unlikely to correlate with prognosis. In patients without tissue hypoxia, no significant differences in survival were observed between the PH medication and PEA/BPA groups, although patients with milder diseases were included in the PH medication group. First, as shown in Additional file 1: Table S7, all hemodynamics at diagnosis indicate improvement predominantly after treatment. The prognosis of PEA is associated with perioperative death and residual PH in the long-term postoperative period [24, 25]. In this study, although perioperative mortality was excluded, 51 patients were treated with selective pulmonary vasodilators due to residual PH, which might have influenced the results. Although 6MWD and DLCO, %pred. were associated with long-term survival in the univariate analysis, we were unable to build a good model in the multivariate analysis using these parameters and PvO_2_. Furthermore, the perioperative mortality was 20% in patients with PVR > 1200 dynes s cm^−5^; our multidisciplinary team discussed whether surgery should be avoided in cases where the PVR is > 1200 dynes s cm^−5^ [26]. Moreover, the surgeon’s technical ability may have influenced the results of the PEA and BPA, suggesting that the levels of these hemodynamic factors at the time of diagnosis did not indicate their prognosis.

Thus, treatment with selective pulmonary vasodilators may be an option for patients with CTEPH without tissue hypoxia. Conversely, PEA or BPA is strongly recommended for patients with tissue hypoxia if there is an indication for PEA or BPA.

In this study, the univariate and multivariate Cox proportional hazards models revealed that PvO_2_ more strongly correlated with prognosis than the other hemodynamic prognostic factors (RAP and CI) in patients with PAH and medically treated CTEPH diagnosed from 1983 to 2018. Recently survival in PAH has improved significantly due to upfront combination therapy becoming the mainstream treatment modality [19]. However, PvO_2_ is still an important prognostic factor in univariate analysis. Surprisingly, PvO_2_ < 35 mmHg was further validated as a prognostic factor in multivariate analyses adjusted by other parameters in the present study. This finding is consistent with that of Khirfan et al.’s study, which was based on ESC/ERS risk stratification and indicated that SvO_2_ was more strongly correlated with prognosis than were thermodilution CI and other parameters in patients with IPAH/HPAH [16]. Tissue oxygenation can be explained using Krogh’s tissue cylinder model [27] (described in Additional file 1: Appendix S1), which forms the theoretical basis for understanding the exchange of oxygen and other solutes between the capillaries and tissues [28]. However, blood sampling at the capillary terminals (termed as the “lethal corner”) is challenging, and tissue hypoxia can be deduced using the mixed venous blood oxygen partial pressure [29, 30]. Based on the oxygen dissociation curve (described in Additional file 1: Appendix S2 and Additional file 5: Fig. S4), SvO_2_ may be normal in a state of alkalosis (e.g., with diuretic use), notwithstanding the presence of tissue hypoxia. Moreover, PvO_2_ can be measured directly using a blood gas analysis. In contrast, SvO_2_ cannot be measured directly using a Swan–Ganz catheter or blood gas analysis; however, it is derived by calculation, which may induce measurement errors. In the present study, logistic regression analyses demonstrated no significant differences between PvO_2_ and SvO_2_ in prognostic ability (data not shown). Thus, PvO_2_ may be more suitable than SvO_2_ for assessing tissue hypoxia.

Survival was significantly poorer in patients with tissue hypoxia at diagnosis than in those without tissue hypoxia in both groups regardless of treatment with selective pulmonary vasodilators. Several studies have conducted survival analyses based on the presence of tissue hypoxia in PH. A prospective study by Kawakami et al. first demonstrated the relative importance of PvO_2_ compared with pulmonary hemodynamics for the prognosis of COPD [10]. PvO_2_ was significantly poorer in non-survivors than in survivors; nonetheless, no significant differences were observed in pulmonary hemodynamics, including the mean PAP and CI, between the groups [10]. Higenbottam et al. reported that SvO_2_, but not CI, was associated with survival in patients with PAH [31, 32]. In the present study, we clarified, for the first time, using PvO_2_ < 35 mmHg as a crucial threshold in patients with PH, that long-term survival was poor in patients with tissue hypoxia.

PvO_2_ is defined by cardiac output, oxygen consumption, hemoglobin content, and PaO_2_. In PAH, the decrease in PvO_2_ may reflect a lower cardiac output and impaired gas exchange. Multiple regression analyses revealed that CI exerted a stronger effect on PvO_2_ than A-aDO_2_ (Additional file 1: Table S4), suggesting that the cause of tissue hypoxia may be related to a lower CI. The decrease in PvO_2_ in CTEPH may also reflect impaired gas exchange and lower cardiac output. However, multiple regression analyses revealed that A-aDO_2_ exerted a greater effect on PvO_2_ than did CI (Additional file 1: Table S5), implying that the cause of tissue hypoxia may be associated with a mismatch in ventilation-perfusion. PAH is characterized by major homogeneous pulmonary vascular remodeling in the pulmonary arterioles (< 0.5 mm in diameter), which may appear as normal or mottled patterns on perfusion scans [33]. However, in CTEPH, the location of the thrombus is heterogeneous on pulmonary perfusion scans. Moreover, hypoperfused areas due to thrombi and hyperperfused areas without thrombi are observed, which are indicative of pulmonary vascular remodeling, similar to PAH. Consequently, a mismatch in ventilation-perfusion may be more notable in CTEPH than in PAH.

PvO_2_ in patients with PAH or CTEPH was not significantly improved by treatment with selective pulmonary vasodilators alone, suggesting that it remains a key prognostic factor even in the current era of multiple combination therapies. However, this finding was inconsistent with the findings of Boucly et al. [7] and Sitbon et al. [34], who suggested that vasodilator treatment improves the SvO_2_ in PAH. This may be explained by the follow-up timing after RHC. A subset of patients received RHC when they were not stabilized or had deteriorated. Particularly, elderly patients with PAH tended to have a smoking history with lower baseline PaO_2_, even without obvious changes in the pulmonary parenchyma on computed tomography. In such cases, ventilation-perfusion mismatching deteriorated with the use of selective pulmonary vasodilators. This finding is consistent with Khirfan et al.’s [35] report describing that older age and a history of smoking are associated with hypoxemia at rest in patients with IPAH/HPAH.

A limitation of the present study is its retrospective design. Furthermore, biases may have occurred in the treatment decisions between the groups with/without tissue hypoxia and among the treatment groups. Additionally, we were unable to propose a model for predicting prognosis in combination with multiple parameters. Some cases with microscopic lung damage that could not be clearly identified as interstitial pneumonia or emphysema on computed tomography were included.

## Conclusions

The present study revealed PvO_2_ as a crucial prognostic factor in PH. The prognostic impact of tissue hypoxia affects different aspects of PAH and CTEPH, reflecting their distinct pathogeneses. Therefore, PvO_2_ can be considered a therapeutic target in patients with PH, warranting further investigation.

## Supplementary Information

**Additional file 1.** (1) **Appendix S1**: Krogh’s Tissue Cylinder Model. (2) **Appendix S2**: Hemoglobin Oxygen Dissociation Curve. (3) **Table S1**. Classification of the enrolled patients with pulmonary arterial hypertension. (4) **Table S2**. Characteristics of patients with PAH stratified by treatment with selective pulmonary vasodilators. (5) **Table S3**. Characteristics of patients with CTEPH stratified by treatment modality. (6) **Table S4**. Coefficients for the CI and A-aDO_2_ affecting PvO_2_ in patients with PAH. (7) **Table S5**. Coefficients for the CI and A-aDO_2_ affecting PvO_2_ in patients with CTEPH. (8) **Table S6**. Hemodynamic and oxygenation parameters before and after treatment with pulmonary vasodilators in patients with PAH. (9) **Table S7**. Hemodynamic and oxygenation parameters before and after treatment in patients with CTEPH. (10) Figure Legends (**Figure S1–S4**).

**Additional file 2: Figure S1.** Selection of study sample.

**Additional file 3: Figure S2.** Kaplan–Meier survival curves stratified by tissue hypoxia in IPAH/HPAH.

**Additional file 4: Figure S3.** Correlations of mixed venous oxygen tension with CI (left) and A-aDO_2_ (right).

**Additional file 5: Figure S4.** Relationship between SvO_2_ and PvO_2_, and the importance of PvO_2_.

## Data Availability

The study database was anonymized, and the study complied with the requirements of the Japanese Ministry of Health, Labour and Welfare. The datasets generated during and/or analyzed during the current study are not publicly available [due to them containing information that could compromise research participant privacy/consent]; however, they are available from the corresponding author (AS) on reasonable request.

## Abbreviations

A-aDO_2_: Alveolar-arterial oxygen gradient
BNP: Brain natriuretic peptide
BPA: Balloon pulmonary angioplasty
CI: Cardiac index
COPD: Chronic obstructive pulmonary disease
CTD: Connective tissue disease
CTEPH: Chronic thromboembolic pulmonary hypertension
DLCO, %pred.: Percent predicted carbon monoxide diffusing capacity
ERS: European Respiratory Society
ESC: European Society of Cardiology
HPAH: Heritable pulmonary arterial hypertension
IPAH: Idiopathic pulmonary arterial hypertension
mPAP: Mean pulmonary arterial pressure
PAH: Pulmonary arterial hypertension
PaCO_2_: Arterial carbon dioxide tension
PaO_2_: Arterial oxygen tension
PEA: Pulmonary endarterectomy
PH: Pulmonary hypertension
PvO_2_: Mixed venous oxygen tension
PVR: Pulmonary vascular resistance
RAP: Right atrial pressure
RHC: Right heart catheterization
SvO_2_: Mixed venous oxygen saturation
WHO: World Health Organization
6MWD: 6-Min walk distance
