# Supplementary material for: Mixed venous oxygen tension is a crucial prognostic factor in pulmonary hypertension: a retrospective cohort study
Source: BMC Pulm Med. 2022 Jul 20;22:282. doi: 10.1186/s12890-022-02073-0 (PMC9301830; doi:10.1186/s12890-022-02073-0)
Supplement: Supplementary file 1 — Additional file 1. (1) Appendix S1: Krogh’s Tissue Cylinder Model. (2) Appendix S2: Hemoglobin Oxygen Dissociation Curve. (3) Table S1. Classification of the enrolled patients with pulmonary arterial hypertension. (4) Table S2. Characteristics of patients with PAH stratified by treatment with selective pulmonary vasodilators. (5) Table S3. Characteristics of patients with CTEPH stratified by treatment modality. (6) Table S4. Coefficients for the CI and A-aDO2 affecting PvO2 in patients with PAH. (7) Table S5. Coefficients for the CI and A-aDO2 affecting PvO2 in patients with CTEPH. (8) Table S6. Hemodynamic and oxygenation parameters before and after treatment with pulmonary vasodilators in patients with PAH. (9) Table S7. Hemodynamic and oxygenation parameters before and after treatment in patients with CTEPH. (10) Figure Legends (Figure S1–S4). [file 12890_2022_2073_MOESM1_ESM.docx]

**SUPPLEMENTAL MATERIAL**

**Mixed Venous Oxygen Tension is a Crucial Prognostic Factor in Pulmonary Hypertension: A Retrospective Cohort study**

Jun Nagata; Ayumi Sekine; Nobuhiro Tanabe; Yu Taniguchi; Keiichi Ishida; Yuki Shiko; Seiichiro Sakao; Koichiro Tatsumi; and Takuji Suzuki

**Appendix S1: Krogh’s Tissue Cylinder Model**

Oxygen supply to the peripheral tissues depends on the partial pressure of oxygen in the tissue capillaries. Oxygen is transferred from the cell surface to intracellular mitochondria by diffusion. During the capillary flow from the arteries to the veins, oxygen is transported from the capillary surface to tissue end components by diffusion due to the pressure gradient. Furthermore, oxygen is utilized by tissues *en route* to being transported from the capillary surface to tissue end components. Subsequently, the partial pressure of oxygen in the tissue exponentially decreases toward the end of the tissue. Moreover, the capillary oxygen content decreases linearly, resulting in a gradual decrease in the partial pressure of oxygen. The tissue supplied by the terminal capillaries has the lowest partial pressure of oxygen, and is therefore vulnerable to hypoxia; accordingly, it is termed the “lethal corner.” Therefore, a decrease in the partial pressure of oxygen at the end of the capillaries below a certain level will hinder the oxygen supply to the tissues in the lethal corner (critical PvO_2_). The partial pressure of oxygen in the venous blood at the end of the capillaries is indicative of the oxygen supply status of the tissue. However, blood sampling at lethal corners is challenging, and tissue hypoxia can be deduced using the mixed venous blood oxygen partial pressure.^1-3^

**Appendix S2: Hemoglobin Oxygen Dissociation Curve**

The relationship between mixed venous oxygen saturation (SvO_2_) and PvO_2_ can be explained by the hemoglobin oxygen dissociation curve (Figure S4). The oxygen dissociation curve depicts the oxygen saturation (SO_2_) bound to hemoglobin and the partial pressure of oxygen (PO_2_) on the vertical and horizontal axes, respectively. Hemoglobin binds tightly to oxygen at high oxygen partial pressures and is rapidly released at low oxygen partial pressures in peripheral tissues.

From the steady state, the dissociation curve shifts to the right with a decrease in pH and/or an increase in the metabolism, body temperature, partial pressure of carbon dioxide, and/or 2,3-bisphosphoglycerate (2,3-DPG) levels (e.g., in pulmonary diseases). Conversely, it shifts to the left with an increase in pH and/or a decrease in the metabolism, body temperature, partial pressure of carbon dioxide, and 2,3-DPG levels (e.g., in pulmonary embolism, ventilation-perfusion ratio mismatch, and diuretic use). Therefore, even if SvO_2_ is normal, tissue hypoxia may be present.

PvO_2_ can be measured directly using a blood gas analysis. In contrast, SvO_2_ cannot be measured directly using a Swan–Ganz catheter or blood gas analysis; however, it is derived by calculation, which may induce measurement errors. Thus, we hypothesized that PvO_2_ may represent a key factor in the identification of tissue hypoxia.

**References**

1.　Krogh A. The number and distribution of capillaries in muscles with calculations of the oxygen pressure head necessary for supplying the tissue. J Physiol. 1919;52:409-15.

2.　Plötz FB, van Lingen RA, Bos AP. Venous oxygen measurements in the inferior vena cava in neonates with respiratory failure. Crit Care. 1998;2:57-60.

3.　Miyamoto K, Kawakami Y. Pathophysiology of tissue hypoxia. Kokyu To Junkan (Respiration and Circulation). 1994;42:437-44 (in Japanese).

| **Table S1.** Classification of the enrolled patients with pulmonary arterial hypertension | |
| --- | --- |
| Classification | N |
| Idiopathic | 56 |
| Hereditary | 5 |
| Drug- and toxin-induced | 1 |
| Associated |  |
| Connective tissue disease | 56 |
| HIV infection | 1 |
| Portal hypertension | 15 |
| Congenital heart disease | 4 |
| Total | 138 |
| HIV = human immunodeficiency virus. Classification of congenital heart disease in four patients; four patients had residual pulmonary hypertension despite interventional or surgical occlusion for atrial septal defect. | |

| **Table S2.** Characteristics of patients with PAH stratified by treatment with selective pulmonary vasodilators | | | |
| --- | --- | --- | --- |
| Variable | Treated | Untreated | *P* |
| N | 104 | 34 |  |
| Age (years) | 51.9±16.6 | 44.8±15.7 | **.003** |
| Sex (F/M) | 82/22 | 31/3 | .105 |
| RAP (mmHg) | 5.8±4.6 | 4.6±2.8 | .404 |
| mPAP (mmHg) | 44.7±11.7 | 49.0±16.2 | .331 |
| CI (L/min/m^2^) | 2.8±0.7 | 2.6±1.0 | .456 |
| PVR (W.U) | 9.6±5.3 | 12.7±8.3 | .142 |
| PaO_2_ (mmHg) | 70.2±13.6 | 75.8±13.5 | **.048** |
| PvO_2_ (mmHg) | 35.7±4.6 | 36.7±5.3 | .288 |
| SvO_2_ (mmHg) | 66.3±8.9 | 68.4±8.7 | .162 |
| A-aDO_2_ (mmHg) | 34.8±14.6 | 29.1±14.5 | **.042** |
| BNP (pg/mL) | 255.6±406.1 | 103.6±138.6 | .190 |
| 6MWD (m) | 367.7±114.3 | 373.7±76.6 | .977 |
| FVC, %pred. (%) | 84.6±19.3 | 88.2±17.2 | .584 |
| DLCO, %pred. (%) | 59.2±20.9 | 55.0±25.3 | .478 |
| WHO functional class Ⅰ/Ⅱ/Ⅲ/Ⅳ | 6/51/42/5 | 0/9/21/4 | **.024** |
| Combination pulmonary vasodilators, n (%) | 57 (54.8%) |  |  |
| ERA, n (%) | 67 (64.4%) |  |  |
| PDE5-I, n (%) | 61 (58.7%) |  |  |
| Prostacyclin, n (%) | 67 (64.4%) |  |  |
| sGCS, n (%) | 7 (6.7%) |  |  |
| Data are presented as mean ± standard deviation or numbers. A-aDO_2_ = alveolar-arterial oxygen gradient; BNP = brain natriuretic peptide; CI = cardiac index; DLCO, %pred. = percent predicted carbon monoxide diffusing capacity; ERA = endothelin receptor antagonists; FVC, %pred. = percent predicted forced vital capacity; mPAP = mean pulmonary arterial pressure; PAH = pulmonary arterial hypertension; PaO_2_ = arterial oxygen tension; PDE5-I = phosphodiesterase type 5 inhibitors; PvO_2_ = mixed venous oxygen tension; PVR = pulmonary vascular resistance; RAP = right arterial pressure; sGCS = soluble guanylate cyclase stimulator; SvO_2_ = mixed venous oxygen saturation; WHO = World Health Organization; W.U = Wood units; 6MWD = 6-min walk distance. | | | |

| **Table S3.** Characteristics of patients with CTEPH stratified by treatment modality | | | | | |
| --- | --- | --- | --- | --- | --- |
| Variable | PEA/BPA | PH medication | Supportive | *P*^a^ | *P*^b^ |
| N | 177 | 66 | 25 |  |  |
| Age (years) | 56.1±12.3 | 61.7±13.1 | 55.0±15.3 | **.003** | **<.001** |
| Sex (F/M) | 125/52 | 55/11 | 15/10 | **.045** | **.044** |
| RAP (mmHg) | 5.5±3.9 | 5.3±4.2 | 4.7±4.2 | .528 | .514 |
| mPAP (mmHg) | 46.0±9.9 | 40.4±10.8 | 40.2±13.9 | **<.001** | **<.001** |
| CI (L/min/m^2^) | 2.6±0.6 | 2.7±0.7 | 2.7±0.7 | .575 | .316 |
| PVR (W.U) | 9.9±3.8 | 8.9±4.7 | 8.9±6.5 | **.013** | .052 |
| PaO_2_ (mmHg) | 58.3±9.3 | 58.0±10.1 | 61.1±13.8 | .698 | .709 |
| PvO_2_ (mmHg) | 33.0±3.5 | 33.3±4.5 | 34.0±5.7 | .129 | .707 |
| SvO_2_ (mmHg) | 62.1±7.0 | 62.6±7.4 | 64.8±10.7 | **.041** | .586 |
| A-aDO_2_ (mmHg) | 46.0±9.8 | 44.0±12.1 | 41.4±16.9 | .146 | .172 |
| BNP (pg/mL) | 234.6±313.7 | 208.4±272.6 | 150.3±257.1 | **.023** | .091 |
| 6MWD (m) | 364.8±95.4 | 346.4±115.1 | 381.8±63.5 | .529 | .299 |
| FVC, %pred. (%) | 94.1±17.4 | 96.3±22.9 | 93.1±17.0 | .283 | .126 |
| DLCO, %pred. (%) | 74.5±19.6 | 75.2±22.3 | 79.0±23.6 | .723 | .091 |
| WHO functional class  Ⅰ/Ⅱ/Ⅲ/Ⅳ | 3/62/103/9 | 0/20/45/1 | 2/12/6/5 | **<.001** | .284 |
| Data are presented as mean ± standard deviation or numbers. A-aDO_2_ = alveolar-arterial oxygen gradient; BNP = brain natriuretic peptide; BPA = balloon pulmonary angioplasty; CI = cardiac index; CTEPH = chronic thromboembolic pulmonary hypertension; DLCO, %pred. = percent predicted carbon monoxide diffusing capacity; FVC, %pred. = percent predicted forced vital capacity; mPAP = mean pulmonary arterial pressure; PaO_2_ = arterial oxygen tension; PEA = pulmonary endarterectomy; PH = pulmonary hypertension; PvO_2_ = mixed venous oxygen tension; PVR = pulmonary vascular resistance; RAP = right arterial pressure; SvO_2_ = mixed venous oxygen saturation; WHO = World Health Organization; W.U = Wood units; 6MWD = 6-min walk distance.  ^a^Comparison among the three groups.  ^b^Comparison between the PEA/BPA and PH medication groups. | | | | | |

| **Table S4.** Coefficients for the CI and A-aDO_2_ affecting PvO_2_ in patients with PAH | | | | | | | |
| --- | --- | --- | --- | --- | --- | --- | --- |
| Model | Unstandardized coefficients | | Standardized coefficients | *t* | *P* | 95% CIv for B | |
|  | B | Std. error | Β |  |  | Lower | Upper |
| Intercept | 31.554 | 1.292 |  | 24.41 | **<.001** | 28.998 | 34.110 |
| CI | 3.224 | 0.358 | 0.522 | 9.01 | **<.001** | 2.516 | 3.932 |
| A-aDO_2_ | -0.135 | 0.018 | -0.435 | -7.51 | **<.001** | -0.170 | -0.099 |
| A-aDO_2_ = alveolar-arterial oxygen gradient; CI = cardiac index; CIv = confidence interval; PAH, pulmonary arterial hypertension; PvO_2_ = mixed venous oxygen tension. | | | | | | | |

| **Table S5.** Coefficients for the CI and A-aDO_2_ affecting PvO_2_ in patients with CTEPH | | | | | | | |
| --- | --- | --- | --- | --- | --- | --- | --- |
| Model | Unstandardized coefficients | | Standardized coefficients | *t* | *P* | 95% CIv for B | |
|  | B | Std. error | Β |  |  | Lower | Upper |
| Intercept | 36.760 | 0.897 |  | 41.00 | **<.001** | 32.852 | 33.439 |
| CI | 2.594 | 0.231 | 0.418 | 11.22 | **<.001** | 4.661 | 6.646 |
| A-aDO_2_ | -0.230 | 0.013 | -0.645 | -17.30 | **<.001** | -9.104 | -7.243 |
| A-aDO_2_ = alveolar-arterial oxygen gradient; CI = cardiac index; CIv = confidence interval; CTEPH = chronic thromboembolic pulmonary hypertension; PvO_2_ = mixed venous oxygen tension. | | | | | | | |

| **Table S6.** Hemodynamic and oxygenation parameters before and after treatment with pulmonary vasodilators in patients with PAH | | | |
| --- | --- | --- | --- |
|  | PAH (N=72) | | |
| Variable | Baseline | After treatment | *P* |
| RAP (mmHg) | 5.4±4.1 | 6.1±4.3 | .169 |
| mPAP (mmHg) | 45.3±12.2 | 40.3±13.4 | **<.001** |
| CI (L/min/m^2^) | 2.8±0.6 | 2.9±0.7 | .063 |
| PVR (W.U) | 9.3±4.9 | 7.3±3.8 | **<.001** |
| PaO_2_ (mmHg) | 71.0±13.6 | 67.3±14.6 | **.025** |
| PaCO_2_ (mmHg) | 36.4±4.1 | 38.9±5.0 | **<.001** |
| PvO_2_ (mmHg) | 36.2±3.9 | 36.0±5.2 | .790 |
| SvO_2_ (mmHg) | 68.0±6.8 | 66.0±8.5 | .065 |
| A-aDO_2_ (mmHg) | 34.7±14.8 | 34.6±16.6 | .982 |
| FVC, %pred. (%) | 88.6±16.0 | 88.2±15.5 | .789 |
| DLCO, %pred. (%) | 61.5±20.3 | 63.3±20.6 | .397 |
| Data are presented as mean ± standard deviation. Post-treatment hemodynamics and oxygenation parameters were adopted from the most recent right heart catheterization data. The mean follow-up time from baseline right heart catheterization was 7.2±7.2 years in patients with PAH.  A-aDO_2_ = alveolar-arterial oxygen gradient; CI = cardiac index; DLCO, %pred. = percent predicted carbon monoxide diffusing capacity; FVC, %pred. = percent predicted forced vital capacity; HPAH = hereditary pulmonary arterial hypertension; IPAH = idiopathic pulmonary arterial hypertension; mPAP = mean pulmonary arterial pressure; PAH = pulmonary arterial hypertension; PaCO_2_ = arterial carbon dioxide tension; PaO_2_ = arterial oxygen tension; PvO_2_ = mixed venous oxygen tension; PVR = pulmonary vascular resistance; RAP = right arterial pressure; SvO_2_ = mixed venous oxygen saturation; W.U = Wood units. | | | |

| **Table S7.** Hemodynamic and oxygenation parameters before and after treatment in patients with CTEPH | | | | | | |
| --- | --- | --- | --- | --- | --- | --- |
|  | With pulmonary vasodilators (N=32) | | | With PEA or BPA (N=138) | | |
| Variable | Baseline | After treatment | *P* | Baseline | After treatment | *P* |
| RAP (mmHg) | 4.5±3.5 | 5.7±2.7 | .087 | 5.3±3.7 | 4.2±3.0 | **<.001** |
| mPAP (mmHg) | 39.5±10.8 | 36.2±13.0 | **.007** | 45.8±10.1 | 25.2±8.3 | **<.001** |
| CI (L/min/m^2^) | 2.7±0.6 | 2.9±1.0 | .444 | 2.6±0.6 | 2.8±0.4 | **<.001** |
| PVR (W.U) | 8.4±4.2 | 7.1±4.5 | **.005** | 9.7±3.7 | 4.0±2.3 | **<.001** |
| PaO_2_ (mmHg) | 59.0±8.2 | 59.4±7.5 | .842 | 58.4±9.1 | 69.1±14.3 | **<.001** |
| PaCO_2_ (mmHg) | 38.1±4.0 | 39.5±4.1 | **.004** | 36.5±3.7 | 40.5±3.7 | **<.001** |
| PvO_2_ (mmHg) | 34.0±3.8 | 34.3±3.5 | .682 | 33.2±3.5 | 36.7±3.9 | **<.001** |
| SvO_2_ (mmHg) | 64.5±6.2 | 63.1±6.7 | .205 | 62.6±6.9 | 67.5±6.7 | **<.001** |
| A-aDO_2_ (mmHg) | 44.0±1.7 | 41.0±10.4 | .111 | 45.6±9.1 | 31.9±15.0 | **<.001** |
| FVC, %pred. (%) | 103.6±19.0 | 99.5±20.2 | **.019** | 93.4±18.4 | 90.6±16.9 | .080 |
| DLCO, %pred. (%) | 76.1±21.5 | 72.3±11.6 | .479 | 75.1±18.5 | 68.6±16.6 | **<.001** |
| Data are presented as mean ± standard deviation. Post-treatment hemodynamic and oxygenation parameters were adopted from the most recent right heart catheterization data. The mean follow-up time from baseline right heart catheterization was 4.8±4.5 years in patients treated with pulmonary vasodilators and 2.7±4.0 years in those treated with PEA or BPA.  A-aDO_2_ = alveolar-arterial oxygen gradient; BPA = balloon pulmonary angioplasty; CI = cardiac index; CTEPH = chronic thromboembolic pulmonary hypertension; DLCO, %pred. = percent predicted carbon monoxide diffusing capacity; FVC, %pred. = percent predicted forced vital capacity; mPAP = mean pulmonary arterial pressure; PaCO_2_ = arterial carbon dioxide tension; PaO_2_ = arterial oxygen tension; PEA = pulmonary endarterectomy; PvO_2_ = mixed venous oxygen tension; PVR = pulmonary vascular resistance; RAP = right arterial pressure; SvO_2_ = mixed venous oxygen saturation; W.U = Wood units. | | | | | | |

**Figure Legends**

Figure S1– Selection of study sample

(A) PAH

(B) CTEPH

BPA = balloon pulmonary angioplasty; CTED = chronic thromboembolic disease; CTEPH = chronic thromboembolic pulmonary hypertension; PAH = pulmonary arterial hypertension; PEA = pulmonary endarterectomy; PH = pulmonary hypertension; PvO_2_ = mixed venous oxygen tension

Figure S2– Kaplan−Meier survival curves stratified by tissue hypoxia in IPAH/HPAH

(A) Group treated with selective pulmonary vasodilators (*P*=.006).

(B) Untreated group (*P*=.011).

IPAH **=** idiopathic pulmonary arterial hypertension; HPAH = heritable pulmonary arterial hypertension.

Figure S3– Correlations of mixed venous oxygen tension with CI (left) and A-aDO_2_ (right)

(A) In pulmonary arterial hypertension (CI: r=0.642, *P*<.001; A-aDO_2_: r=-0.549, *P*<.001)

(B) In chronic thromboembolic pulmonary hypertension (CI: r=0.470, *P*<.001; A-aDO_2_: r=-0.678, *P*<.001)

PvO_2_ = mixed venous oxygen tension; CI = cardiac index; and A-aDO_2_ = arterial oxygen gradient.

Figure S4– Relationship between SvO_2_ and PvO_2_, and the importance of PvO_2_

The figure depicts the hemoglobin oxygen dissociation curve. If the curve shifts to the left, the patients may experience tissue hypoxia (PvO_2_ <35 mmHg), despite a normal SvO_2_.

PaO_2_ = arterial oxygen tension; SaO_2_ = arterial oxygen saturation; SvO_2_ = mixed venous oxygen saturation; PvO_2_ = mixed venous oxygen tension
