# Supplementary figures and images for: Mixed venous oxygen tension is a crucial prognostic factor in pulmonary hypertension: a retrospective cohort study
Source: BMC Pulm Med. 2022 Jul 20;22:282. doi: 10.1186/s12890-022-02073-0 (PMC9301830; doi:10.1186/s12890-022-02073-0)

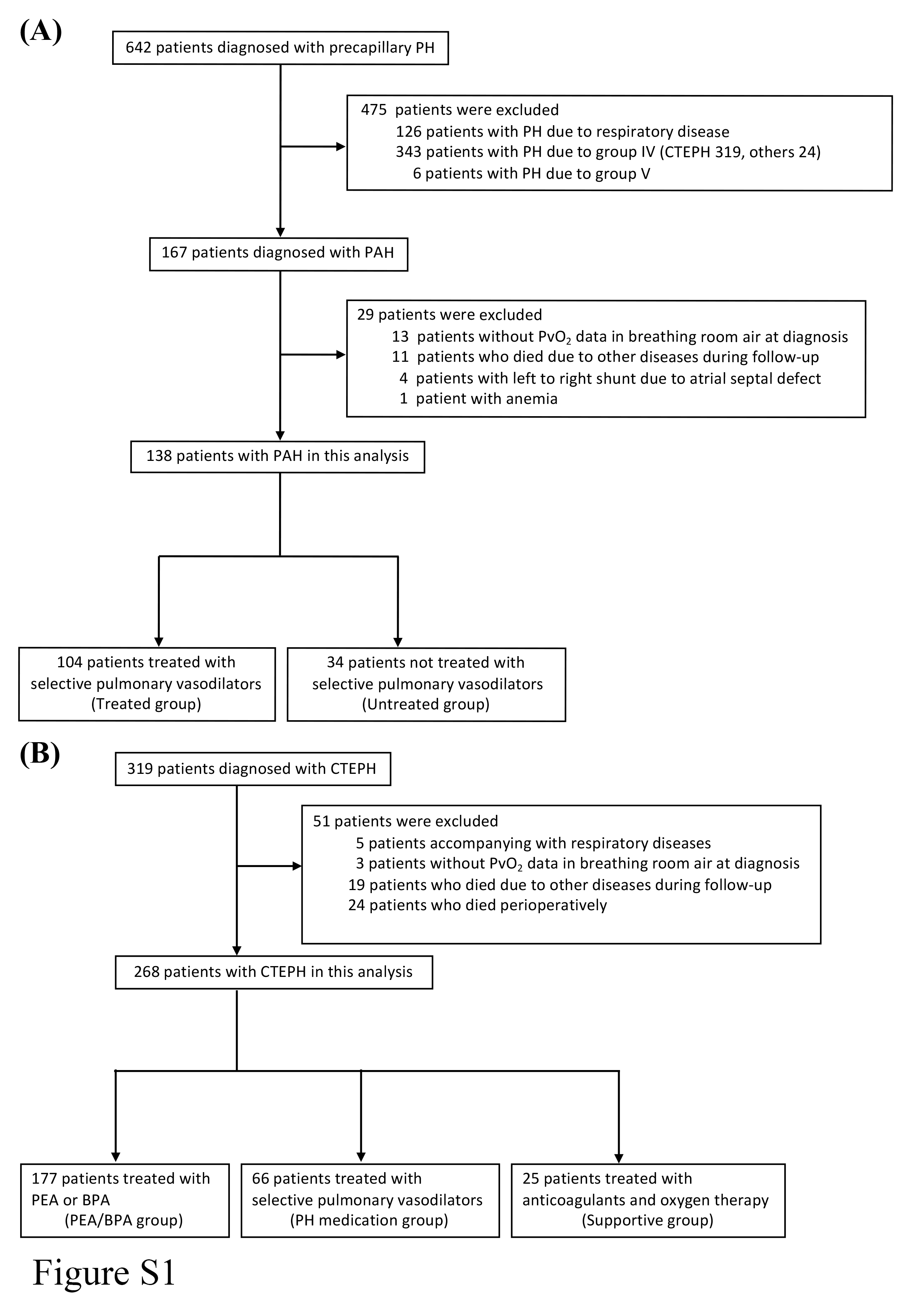

Supplement: Supplementary file 2 — Additional file 2: Figure S1. Selection of study sample. [file 12890_2022_2073_MOESM2_ESM.jpg]

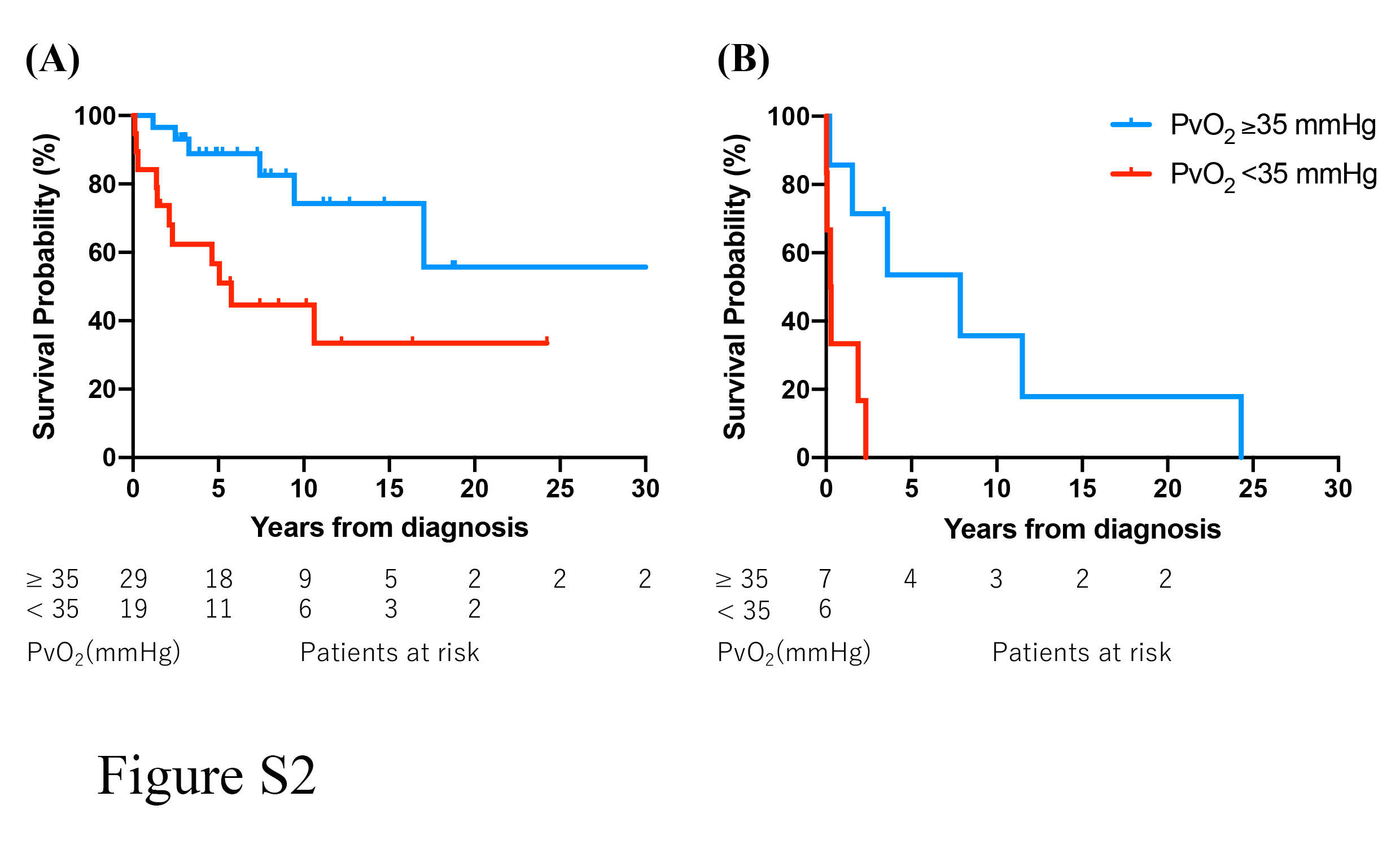

Supplement: Supplementary file 3 — Additional file 3: Figure S2. Kaplan–Meier survival curves stratified by tissue hypoxia in IPAH/HPAH. [file 12890_2022_2073_MOESM3_ESM.jpg]

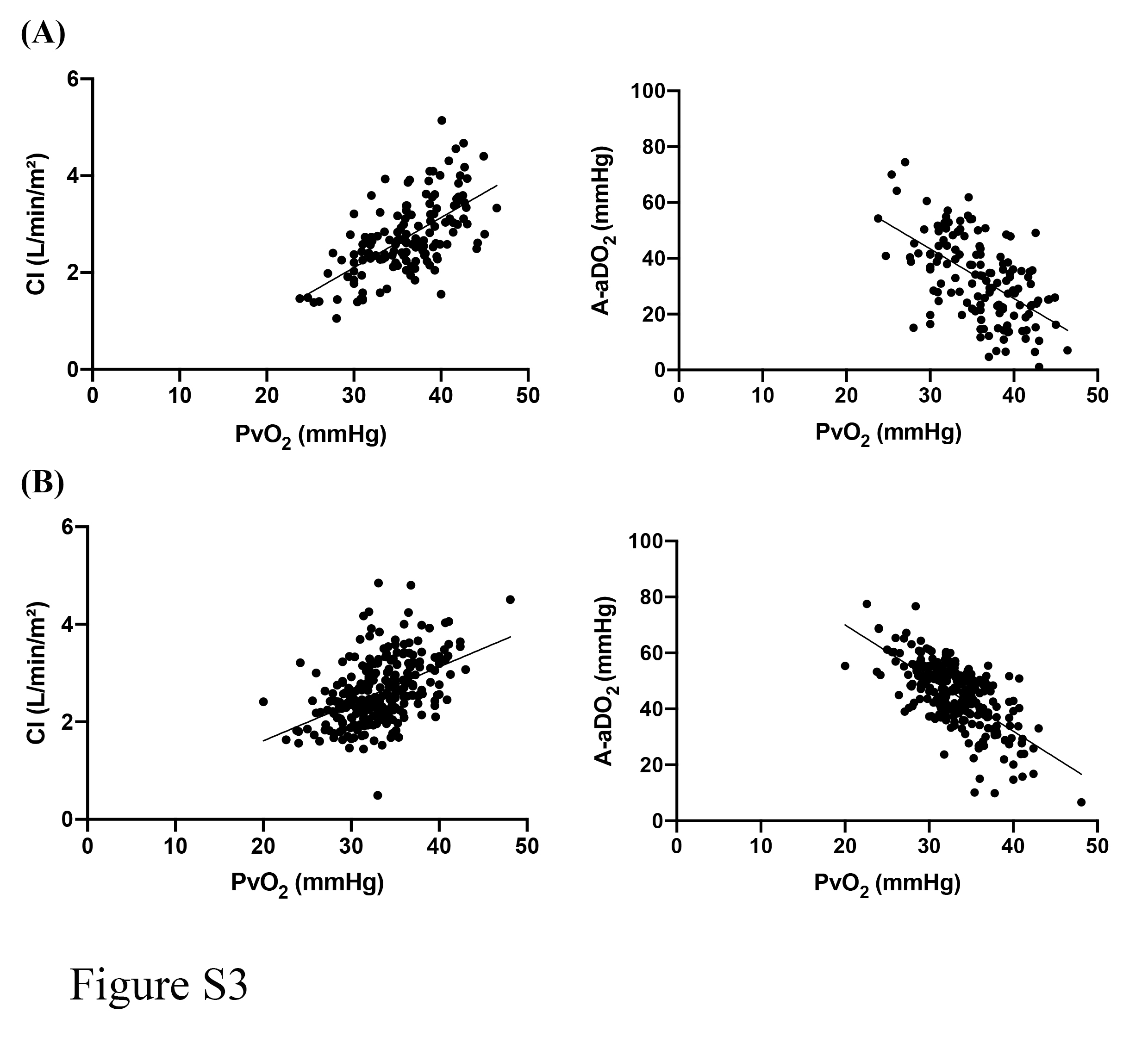

Supplement: Supplementary file 4 — Additional file 4: Figure S3. Correlations of mixed venous oxygen tension with CI (left) and A-aDO2 (right). [file 12890_2022_2073_MOESM4_ESM.jpg]

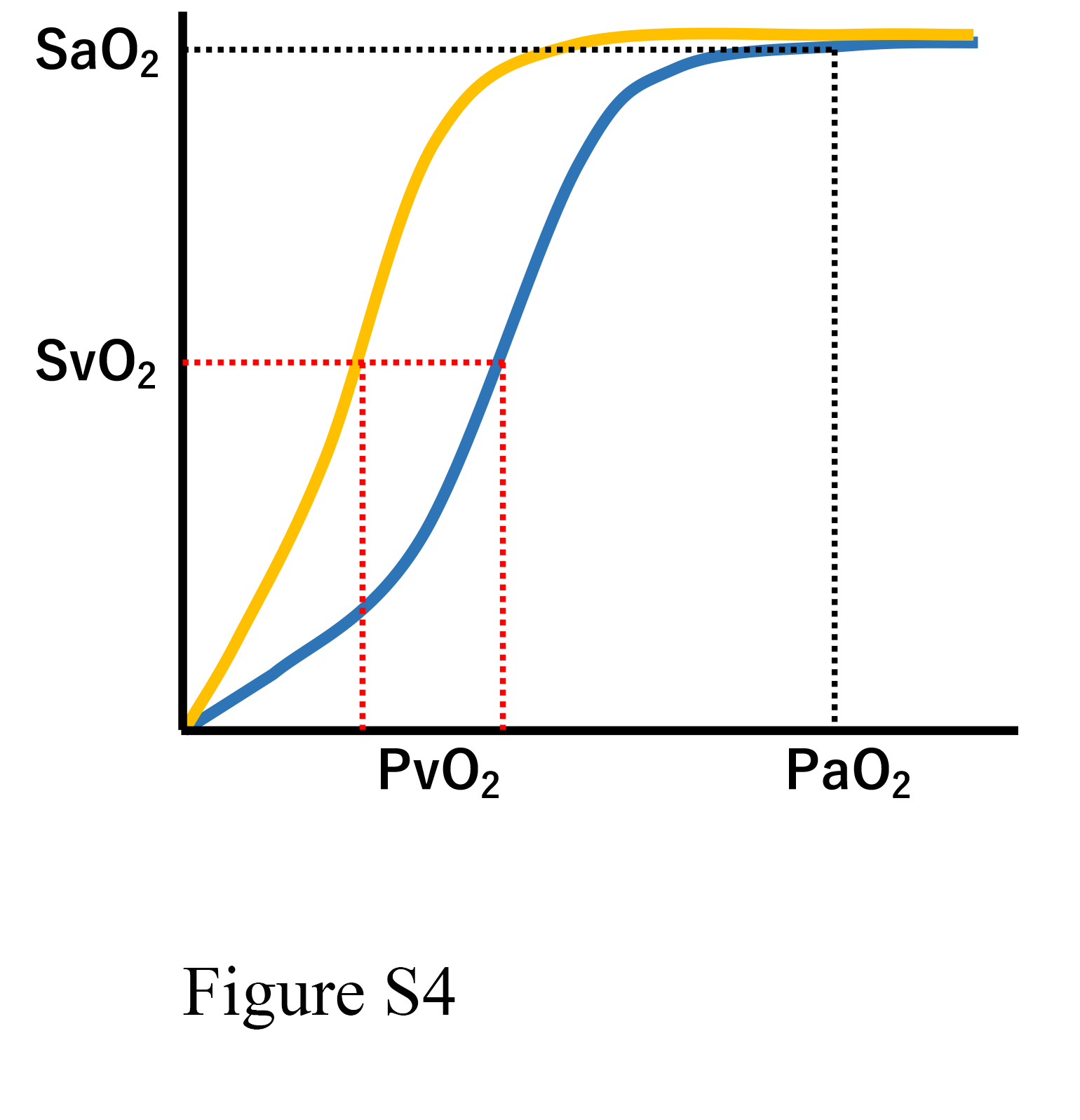

Supplement: Supplementary file 5 — Additional file 5: Figure S4. Relationship between SvO2 and PvO2, and the importance of PvO2. [file 12890_2022_2073_MOESM5_ESM.jpg]
